# Fatty Acid Heterogeneity in Colorectal Cancer: From Food Matrices to Targeted Interventions

**DOI:** 10.3390/cancers18183059

**Published:** 2026-09-21

**Authors:** María Jesús Vera, Camila Farias, Yasna Muñoz, Nicolás Ortiz-López, Héctor R. Contreras, Rodrigo Valenzuela

**Affiliations:** 1 Department of Nutrition, Faculty of Medicine, University of Chile, Santiago 8380000, Chile; maria.vera@inta.uchile.cl (M.J.V.); camila.farias.c@uchile.cl (C.F.); nicolas.ortiz@ug.uchile.cl (N.O.-L.); 2 Laboratory of Cellular and Molecular Oncology, Department of Basic and Clinical Oncology, Faculty of Medicine, University of Chile, Santiago 8320328, Chile; hcontrer@uchile.cl; 3 Center for Cancer Prevention and Control (CECAN), Santiago 8380453, Chile; 4 Section of Gastroenterology, Department of Internal Medicine, University of Chile Clinical Hospital, Santiago 8380453, Chile

**Keywords:** colorectal cancer, polyunsaturated fatty acids, n-3 fatty acids, fatty acid metabolism, dietary patterns, lipidomics, cancer prevention

## Abstract

Fatty acids are dietary exposures, membrane components, signaling precursors, and products of endogenous metabolism. Their relationship with colorectal cancer (CRC) is therefore difficult to interpret because dietary intake, circulating and erythrocyte biomarkers, tumor tissue, genetically predicted levels, and interventions represent different biological phenomena. This structured narrative review integrates these domains to explain heterogeneous associations with CRC risk, precursor lesions, progression, and clinical outcomes. Human evidence does not support a simple classification in which saturated fatty acids (SFAs) and n-6 polyunsaturated fatty acids (n-6 PUFAs) are uniformly harmful, monounsaturated fatty acids (MUFAs) are neutral, and n-3 polyunsaturated fatty acids (n-3 PUFAs) universally protective. Associations vary by individual fatty acid, food source, biological matrix, endogenous desaturation and elongation, tumor site, sex, stage, and study design. Circulating n-3 PUFA biomarkers show several inverse associations, including prospective UK Biobank evidence, but relationships may be nonlinear and site- and sex-specific. Tumor lipid profiles demonstrate metabolic remodeling but cannot establish causal exposure. Mendelian randomization provides complementary evidence but is sensitive to pleiotropy and metabolic correlation. Intervention studies suggest benefits for selected perioperative, inflammatory, immune, or nutritional outcomes, whereas evidence for adenoma prevention, CRC recurrence, or survival remains limited. Interpretation should therefore consider the distinct evidence domains rather than total fat classes or the n-6/n-3 PUFA ratio alone.

## Simple Summary

Fatty acids are essential components of the diet and participate in cell structure, metabolism, inflammation, and signaling. However, studies examining their relationship with colorectal cancer have produced heterogeneous results because they assess different aspects of fatty acid exposure, including dietary intake, blood biomarkers, tumor tissue, genetic variation, and nutritional interventions. This review integrates complementary forms of human evidence to clarify what each approach can and cannot demonstrate. Current evidence does not support classifying broad groups of fatty acids as uniformly harmful or protective. Although selected n-3 fatty acids show potentially favorable associations in some settings, available data remain insufficient to recommend routine supplementation, a specific dose, or a target n-6 to n-3 ratio for colorectal cancer prevention or treatment. By distinguishing dietary exposure from circulating, tissue, genetic, and intervention evidence, this review provides a clearer framework for interpretation, identifies knowledge gaps, and may guide the design of future relevant studies.

## 1. Introduction

Colorectal Cancer (CRC) is a major cause of cancer morbidity and mortality worldwide, and its geographic variation reflects the combined influence of inherited susceptibility, environmental exposures, and lifestyle factors [1,2,3]. Diet is central to current prevention frameworks, although the contribution of individual nutrients is best interpreted within food sources and dietary patterns rather than in isolation [4,5]. Fatty acids are especially relevant because they are not only energy substrates but also structural components of cell membranes and precursors of signaling molecules that regulate survival, proliferation, immune responses, inflammation, and oxidative processes [6]. This biological versatility has encouraged extensive research on saturated fatty acids (SFAs), monounsaturated fatty acids (MUFAs), and polyunsaturated fatty acids (PUFAs) in CRC [7,8]. However, the resulting literature is heterogeneous, and apparently conflicting findings often arise because studies measure fundamentally different aspects of fatty-acid exposure and metabolism rather than the same biological construct.

Dietary intake, circulating biomarkers, erythrocyte composition, tumor tissue, genetic proxies, and supplementation trials should therefore not be interpreted as interchangeable evidence. Dietary assessment reflects habitual food choice but is affected by recall errors, food matrix, cooking methods, and total energy intake. Plasma and serum integrate recent or habitual intake with intestinal absorption, hepatic metabolism, adipose-tissue mobilization, de novo lipogenesis, and desaturase and elongase enzyme activity [9,10]. Erythrocytes generally represent a longer exposure window, yet their fatty-acid composition is still metabolically modified. In contrast, a resected tumor reflects events that occur after neoplastic transformation, including selective fatty-acid uptake, de novo synthesis, membrane remodeling, proliferative demand, hypoxia, inflammation, stromal composition, and treatment-related adaptation [9,11,12,13]. Mendelian randomization (MR) estimates the effect of genetically influenced metabolic traits over the life course and can reduce some forms of confounding and reverse causation, but its interpretation depends on instrument validity, pleiotropy, ancestry, and correlations among metabolites that share FADS and elongase pathways [14,15,16,17,18,19]. Finally, nutritional interventions test defined formulation, dose, duration, and clinical setting. A perioperative change in C-reactive protein or interleukin-6 is therefore not equivalent to CRC prevention, reduced adenoma recurrence, or improved survival. Recognizing these differences is essential before comparing effect estimates across studies.

The central problem is consequently not whether one broad lipid class is simply beneficial or harmful, but why different methods of studying fatty acids produce divergent conclusions. A dietary association may be null while a circulating biomarker is associated with risk, and a fatty acid may be enriched in tumor tissue without having caused the tumor. These patterns can be biologically coherent rather than contradictory.

Previous reviews have commonly examined individual fatty-acid classes, n-3 PUFA supplementation, or isolated exposure domains. The present review instead integrates dietary intake and food sources, circulating and erythrocyte biomarkers, tumor-tissue lipidomics, endogenous fatty-acid metabolism, genetic and MR analyses, precursor-lesion studies, and nutritional interventions. The objective is to explain heterogeneous associations with CRC risk, anatomical subsite, molecular progression, treatment-related outcomes, and survivorship, while distinguishing observation, causal inference, mechanistic plausibility, and clinical applicability. Particular attention is given to the fact that individual fatty acids within the same family may behave differently, that food source and nutrient substitution modify the interpretation of dietary SFAs and MUFAs, that dihomo-gamma-linolenic acid (C20:3n-6, DGLA) and arachidonic acid (C20:4n-6, ARA) are not biologically equivalent n-6 PUFA exposures, and that the n-6/n-3 PUFA ratio can obscure which fatty acid actually drives an association. This framework also addresses the translational questions most relevant to nutrition and oncology: which findings can currently inform prevention or patient care, which remain exploratory, and which require prospective validation?

## 2. Methodology

This study was conducted as a structured narrative review designed to integrate complementary domains of human evidence rather than to generate a single pooled effect estimate. The methodology was structured to improve transparency in identification of the literature, evidence classification, and interpretation across study designs.

Literature searches were conducted in PubMed/MEDLINE and updated through 13 January 2026. Search terms combined colorectal neoplasia-related terms (“colorectal cancer”, “colon cancer”, “rectal cancer”, and “colorectal adenoma”) with terms related to fatty-acid exposure and metabolism, including “fatty acids”, “saturated fatty acids”, “monounsaturated fatty acids”, “polyunsaturated fatty acids”, “omega-3”, “omega-6”, “EPA”, “DHA”, “arachidonic acid”, “linoleic acid”, and “alpha-linolenic acid”. The search was not conducted separately for each evidence domain. Rather, studies identified through these fatty-acid-related searches were subsequently classified according to the type of evidence they provided. The resulting evidence domains included dietary intake and food sources, circulating and erythrocyte fatty acids, tumor tissue and lipidomic remodeling, genetic and Mendelian-randomization evidence, precursor lesions and chemoprevention, interventions after CRC diagnosis, and diet–microbiota–bile-acid interactions. These categories were therefore used as an organizational framework for evidence synthesis rather than as independent search strategies. 

The primary search focused on human studies published from January 2021 through January 2026. Selected earlier publications were additionally included when they were considered particularly relevant to the interpretation of the current evidence. These studies were incorporated only when they provided foundational information on fatty-acid metabolism, represented landmark human studies or clinical trials, or were necessary to contextualize and explain findings identified in contemporary literature.

Eligible evidence included prospective cohorts, case-control studies, cross-sectional biomarker or lipidomic studies, tumor-adjacent tissue studies, studies of colorectal precursor lesions, randomized controlled trials, Mendelian-randomization analyses, and systematic reviews or meta-analyses reporting human CRC-related outcomes. Experimental studies were used only to provide mechanistic context and were not considered evidence of clinical efficacy. The identified studies were subsequently organized into the following evidence domains: (1) dietary intake and food sources, (2) circulating and erythrocyte fatty acids, (3) tumor tissue and lipidomic remodeling, (4) genetic and Mendelian-randomization evidence, (5) precursor-lesion and chemoprevention evidence, (6) interventions after CRC diagnosis, and (7) diet–microbiota–bile-acid interactions.

Evidence was interpreted according to the biological construct captured by each design, the temporality of exposure assessment, susceptibility to confounding or reverse causation, and the clinical relevance of the reported endpoint. Observational associations were not interpreted as causal; post-diagnostic tissue profiles were not treated as etiological exposures, and changes in inflammatory, immune, or nutritional surrogate outcomes were not considered evidence of reduced CRC recurrence or improved survival. No formal risk-of-bias or certainty-of-evidence assessment was performed.

## 3. Evidence Synthesis

### 3.1. Biological Framework of Fatty Acid Metabolism

**Figure 1:**
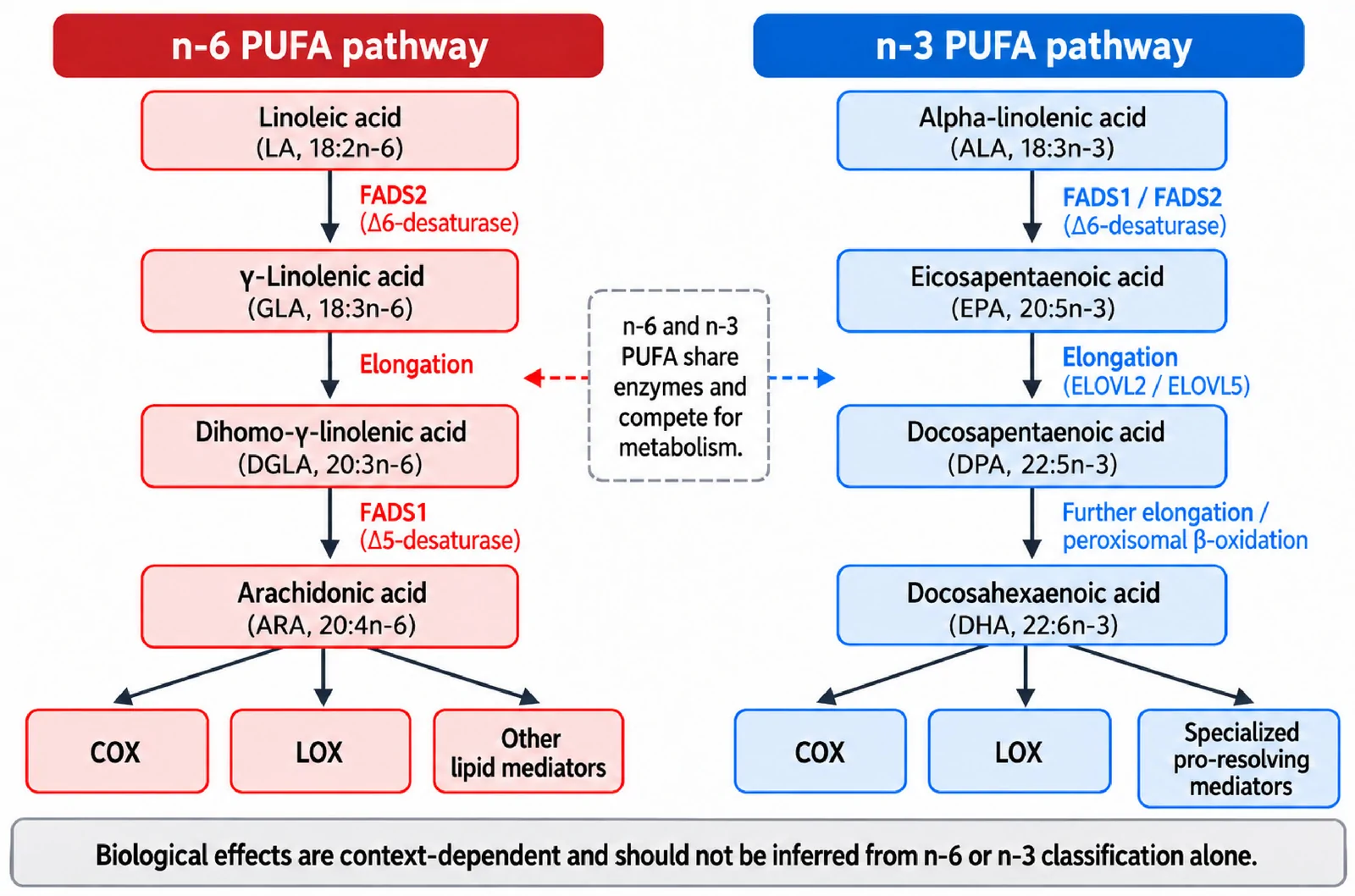
Polyunsaturated fatty-acid metabolism and selected pathways relevant to colorectal cancer. The figure illustrates the shared desaturation and elongation pathways connecting n-6 and n-3 fatty acids and their downstream metabolism into bioactive lipid mediators. ARA, DGLA, EPA, and DHA can generate distinct mediator profiles through cyclooxygenase, lipoxygenase, and related pathways. Their biological effects depend on substrate availability, enzyme expression, cellular and tissue context, inflammatory state, and disease stage. Accordingly, the figure should not be interpreted as indicating that n-6 PUFAs are uniformly harmful or that n-3 PUFAs are uniformly protective. Abbreviations: PUFA, polyunsaturated fatty acid, LA, linoleic acid, ALA, alpha-linolenic acid, GLA, gamma-linolenic acid, DGLA, dihomo-gamma-linolenic acid, ARA, arachidonic acid, EPA, eicosapentaenoic acid, n-3 DPA, docosapentaenoic acid, DHA, docosahexaenoic acid, FADS1, fatty acid desaturase 1, FADS2, fatty acid desaturase 2, COX, cyclooxygenase, LOX, lipoxygenase.

A biologically coherent interpretation requires explicit separation of exogenous exposure from endogenous lipid metabolism. Fatty acids may be obtained from foods, synthesized de novo, elongated, desaturated, incorporated into membrane phospholipids, stored in neutral lipids, oxidized for energy, peroxidized, or converted into bioactive mediators [6,9,11,12]. These processes occur simultaneously; so, the concentration of a fatty acid in blood or tumor tissue represents the net result of several metabolic fluxes rather than a direct readout of intake. Fatty acid synthase (FASN) supports de novo generation of palmitate, whereas stearoyl-CoA desaturase-1 (SCD1) converts saturated acyl-CoAs toward MUFAs and contributes to membrane-lipid remodeling, both pathways can be altered in malignant cells [11,20,21]. Increased endogenous synthesis can therefore raise or redistribute SFAs and MUFAs even when dietary exposure is unchanged. This distinction is particularly relevant in proliferating tumor cells, where lipid supply must support membrane biogenesis, signaling, energy metabolism, and adaptation to the microenvironment [9,11,12]. It also explains why dietary SFA or MUFA associations should not be used to infer the meaning of the same lipid species measured after diagnosis in tumor tissue. The PUFA pathways introduce an additional layer of complexity. Essential linoleic acid (C18:2n-6, LA) and alpha-linolenic acid (C18:3n-3, ALA) enter shared desaturation and elongation pathways whose activity depends on substrate availability, genetics, hepatic metabolism, and competition for common enzymes [19,22,23,24]. For n-6 PUFAs, the pathway should be represented explicitly as LA to gamma-linolenic acid (C18:3n-6, GLA), to dihomo-gamma-linolenic acid (C20:3n-6, DGLA), and then to ARA. FADS2 participates in the initial desaturation step, elongases extend the carbon chain, and FADS1 catalyzes conversion of DGLA to ARA [19,23,24] (Figure 1).

The n-3 PUFA pathway begins with ALA and proceeds through elongation and desaturation toward eicosapentaenoic acid (C20:5n-3, EPA), docosapentaenoic acid (C22:5n-3, n-3 DPA), and docosahexaenoic acid (C22:6n-3, DHA), involving FADS1/FADS2 and and elongases such as ELOVL2 and ELOVL5 [19,22,23,24]. Because n-6 and n-3 PUFA substrates share enzymes, concentrations of individual intermediates reflect pathway flux and cannot be interpreted solely as dietary exposure. DGLA is a particularly important example. Higher DGLA does not necessarily indicate greater functional ARA exposure; DGLA may accumulate when FADS1-mediated conversion to ARA is reduced. Consequently, DGLA and ARA should not be grouped as equivalent n-6 exposures, and the ARA/DGLA ratio can provide information related to delta-5-desaturase activity [18,19,25]. This distinction helps reconcile observations in which erythrocyte DGLA is inversely associated with advanced adenomas [26] while genetic studies of other n-6 pathway components show different directions of association [15,18]. Downstream metabolism further separates chemical class from biological action. ARA can be released from membrane phospholipids and converted through cyclooxygenase and lipoxygenase pathways into eicosanoid mediators involved in inflammation, vascular responses, proliferation, and tumor-associated signaling [25,26,27]. EPA and DHA can compete for parts of this enzymatic machinery and serve as precursors for lipid mediators involved in inflammatory resolution [7,8,22,28,29,30]. The balance of these products depends not simply on dietary n-6/n-3 PUFA intake but on substrate availability, enzyme expression, membrane composition, cell type, and inflammatory context. Membrane incorporation can also alter fluidity and receptor organization, whereas oxidation and lipid peroxidation may have different consequences according to antioxidant capacity and tumor metabolic state. For this reason, a high concentration of an individual PUFA is not intrinsically protective or harmful. The biologically relevant question is where the fatty acid was measured, how it was generated, which metabolic products predominate, and at what stage of colorectal carcinogenesis or treatment the measurement was obtained.

### 3.2. Dietary Fatty Acids, Food Sources, and CRC Risk

Earlier concerns about SFA were informed partly by cardiometabolic and inflammatory literature [31,32,33,34]. Dietary evidence, however, does not support a simple model in which all SFAs are independently harmful and all MUFAs are neutral. In the Shanghai Men’s Health Study, SFA and MUFA intake were not significantly associated with CRC risk, and these findings were consistent with those of a meta-analysis [35]. In the Black Women’s Health Study, total SFAs and MUFAs were also not associated with CRC, although unprocessed red meat was associated with higher risk [36]. This distinction is important because the risk attributed to red or processed meat cannot automatically be assigned to its SFA content: heme iron, processing, cooking-derived compounds, sodium, and the broader dietary pattern also contribute to the exposure [37,38,39]. Dietary-pattern analyses reinforce this point. An Iranian case–control study identified patterns rich in unhealthy fats and animal foods that were associated with CRC [40], while in the UK Biobank study, a pattern characterized by SFAs, free sugars, red and processed meat, and low fiber was associated with overall and rectal cancer [41]. These findings support interpretation at the level of food source, substitution, and dietary matrix rather than isolated SFA grams [39,42,43,44]. MUFAs should be considered similarly: olive oil, nuts, and MUFAs derived from meat are not nutritionally equivalent exposures [5,20,45,46].

Taken together, the observational dietary evidence provides limited support for an independent class-wide association between total SFA or MUFA intake and CRC risk. Prospective cohort findings are predominantly null or heterogeneous, whereas associations identified for dietary patterns or specific food sources cannot isolate the effect of fatty acids from correlated exposures such as fiber intake, red and processed meat, food processing, or overall dietary quality. In addition, measurement errors inherent to dietary assessment and residual confounding may attenuate or distort individual fatty-acid associations. Therefore, these findings are more appropriately interpreted at the level of food source, dietary pattern, and nutrient substitution than as evidence of an intrinsic harmful or protective effect of SFAs or MUFAs. A summary of recent evidence is presented in Table 1. 

### 3.3. PUFA Intake: Individual Fatty Acids and Sources

PUFA intake is equally heterogeneous and should be analyzed by individual fatty acid, food source, and population context rather than by a universal n-6 PUFA versus n-3 PUFA dichotomy [23,24,47]. In the Japan Collaborative Cohort Study, ALA intake was inversely associated with distal colon cancer, whereas marine n-3 PUFA were not significantly associated with overall CRC or all anatomical subsites [48]. A dose-response meta-analysis also suggested inverse associations for ALA exposure [49]. In the Black Women’s Health Study, baked fish intake was associated with lower CRC risk and higher n-3 PUFA intake with lower proximal colon cancer risk, whereas fried fish did not show the same pattern [50]. This difference illustrates that fatty acid intake is embedded within preparation method, accompanying nutrients, and the food matrix rather than being delivered as an isolated exposure. A case-control study reported inverse associations for dietary LA and ALA [51], and the meta-analysis by Lu et al. found lower CRC risk with higher EPA, DHA, and n-3 DPA intake in selected comparisons, while results for total MUFAs remained null and SFA findings were heterogeneous [52]. These findings do not justify treating all n-6 fatty acids as pro-tumorigenic. LA, GLA, DGLA, and ARA occupy different positions in the pathway and can therefore produce different epidemiological signals. Likewise, the n-6/n-3 PUFA ratio should not be interpreted as a universal causal marker because the same numerical ratio can result from biologically different combinations of LA, DGLA, ARA, EPA, and DHA. Substitution also matters; replacing SFAs with PUFAs is not equivalent to adding PUFAs on top of an unchanged dietary pattern, and interpretation should consider the nutrient or food displaced [39,40,41,42]. Molecular analyses require similar caution. The association reported between high total PUFA intake and *KRAS*-mutated tumors does not demonstrate that PUFAs cause *KRAS* mutation, and *KRAS* is better understood as a driver of adenoma progression rather than an initiating event equivalent to APC [53,54].

Overall, observational studies provide suggestive but not definitive evidence of inverse associations for selected PUFA exposures, particularly ALA and some long-chain n-3 fatty acids. However, these associations are not uniform across individual fatty acids, anatomical subsites, populations, or food sources. Case-control studies remain susceptible to recall and selection bias, while even prospective dietary studies are limited by measurement error and residual confounding. Moreover, associations observed for fish intake cannot be attributed exclusively to EPA or DHA because fish consumption also captures preparation methods, dietary substitution, and other nutritional characteristics. Thus, the observational evidence supports further investigation of specific fatty acids rather than a general classification of n-3 PUFAs as protective or n-6 PUFAs as harmful (Table 2).

### 3.4. Circulating and Erythrocyte Fatty Acids

Circulating and erythrocyte fatty acids provide objective biological measurements but should not be treated as direct measures of dietary intake. Plasma and serum integrate recent or habitual consumption with intestinal absorption, endogenous synthesis, hepatic lipid handling, desaturase and elongase ezyme activity, adipose-tissue mobilization, and the size and composition of the lipid fraction in which fatty acids are expressed [10]. Erythrocyte membranes generally represent a longer exposure window, yet they are also shaped by metabolic conversion and membrane turnover. This distinction helps explain why dietary and blood-based analyses can produce different directions of association. For SFAs, Wu et al. reported that total serum SFAs were positively associated with CRC in a Chinese case-control study, whereas very-long-chain SFAs were inversely associated [55]. A small Korean case-control analysis likewise identified higher concentrations of several circulating SFAs in CRC cases together with altered *PTGS2* expression [56]. These findings are better interpreted as disease-associated metabolic profiles than as proof that dietary SFAs caused CRC, particularly because post-diagnostic sampling is vulnerable to reverse causation. In contrast, large dietary cohorts have generally shown null or heterogeneous total-SFA associations [35,36,52]. Accordingly, biomarker studies should also be interpreted according to temporality. Fatty-acid profiles measured after CRC diagnosis are particularly difficult to distinguish from disease-related metabolic remodeling, whereas measurements obtained prospectively before diagnosis provide stronger temporal evidence. Nevertheless, prospective biomarkers still represent integrated metabolic phenotypes rather than isolated dietary exposures and therefore do not, by themselves, establish that modifying dietary intake would reproduce the observed association.

The apparent discordance is therefore biologically plausible rather than necessarily contradictory. Biomarker interpretation should also distinguish individual fatty acids from totals, serum or plasma from erythrocytes, and absolute concentrations from percentages of total fatty acids. Because percentage measures are compositional, an increase in one fatty acid can mathematically reduce the relative proportion of another even when its absolute concentration is unchanged. In precursor-lesion research, erythrocyte PUFAs were largely unassociated with conventional adenomas or serrated polyps, although DGLA showed an inverse association with advanced adenomas [26]. Together, these studies support analysis at the level of individual fatty acids and metabolic pathways rather than broad circulating SFA, MUFA, n-6 PUFA, or n-3 PUFA categories. Aldoori et al. evaluated baseline plasma total n-3 PUFA and DHA in 234,598 UK Biobank participants followed for a mean of 13.4 years, during which 2602 incident CRC cases occurred [57]. Higher tertiles showed modest inverse associations for both total n-3 PUFA and DHA [57]. The DHA association was nonlinear, with evidence of a plateau at higher concentrations. Subsite analyses suggested a stronger inverse association for proximal colon cancer, and sex-stratified analyses showed the association more clearly in men than in women [57]. These results strengthen prospective biomarker evidence while simultaneously demonstrating why a single statement that “higher n-3 PUFA is protective” is inadequate. The exposure was assessed via a plasma biomarker, the dose response was not simply linear, and the magnitude of association varied according to anatomical site and patient characteristics. Thus, although prospective biomarker studies reduce concerns regarding reverse causation compared with post-diagnostic case-control analyses, the modest magnitude of the associations, nonlinearity, subsite and sex heterogeneity, and dependence on the lipid metric measured argue against defining a universal protective concentration or clinically relevant threshold for n-3 fatty acids.

These findings should be interpreted alongside the broader prospective and meta-analytic literature. Kim et al. previously reported an inverse pooled association between higher blood n-3 PUFA levels and CRC in prospective studies [29], while Lu et al. showed that dietary and circulating results were not fully concordant across individual fatty acids [52]. A separate UK Biobank cohort analysis of more than 250,000 participants evaluated plasma n-3 and n-6 PUFA percentages against overall and site-specific cancer incidence [58]. It found small inverse associations for several malignancies, including an inverse association between higher n-3 PUFA and colon cancer; higher n-6 PUFA percentages also showed inverse associations for several cancer sites [58]. Although that analysis was not designed specifically around CRC and reported relative fatty-acid measures, it directly challenges the assumption that a higher circulating n-6 PUFA percentage is intrinsically harmful. Differences between studies can arise from the lipid fraction measured, absolute versus relative expression, timing of sampling, and preclinical metabolic change. Heterogeneity by sex, metabolic status, proximal versus distal colon, and rectal site may also reflect differences in fatty-acid metabolism and tumor biology. Consequently, a circulating fatty acid should be viewed as an integrated metabolic phenotype [57,58,59,60].

### 3.5. Tumor-Tissue Fatty Acids and Lipidomic Remodeling

Tumor-tissue composition addresses a fundamentally different question from dietary or circulating risk studies. A fatty acid measured after diagnosis may reflect selective uptake, de novo synthesis, membrane remodeling, proliferative demand, hypoxia, inflammation, stromal or immune-cell composition, and treatment-related adaptation [9,11,12,13]. Comparative analyses of CRC tissue and adjacent mucosa demonstrate substantial lipid remodeling. Juloski et al. reported higher stearic acid and total SFAs in tumor tissue, whereas oleic acid (C18:1n-9, OA) and total MUFAs were higher in adjacent non-tumor mucosa [61]. Other work has shown preferential uptake of PUFAs by CRC cells and differences in ARA, EPA, DHA, LA, and ALA between tumor and non-tumor compartments [13,62]. These patterns are compatible with altered membrane requirements and metabolic adaptation, but they do not imply that the fatty acids enriched in the tumor tissue increased CRC risk. The cross-sectional and post-diagnostic nature of these comparisons therefore prevents determination of whether the observed lipid differences preceded carcinogenesis or emerged because of tumor development.

The same caution applies to lipidomic profiling and proposed fatty-acid-based translational strategies [21,63,64]. Serum metabolomics and lipidomics have distinguished CRC, adenoma, and control groups using panels that include long-chain n-3 and n-6 PUFA [65], while extracellular-vesicle studies have identified altered degrees of lipid unsaturation across colorectal disease states [66]. Given the limited sample sizes and lack of external validation, these findings should be described as candidate or exploratory lipidomic signatures rather than established diagnostic or prognostic biomarkers [65,66,67]. Genetic variation in lipid-metabolism pathways, including *FASN*-related variants associated with progression-free survival during bevacizumab-based therapy, is similarly hypothesis-generating with regard to tumor metabolic dependency and precision oncology [67].

### 3.6. Genetic and Mendelian Randomization Evidence

MR provides a complementary approach to causal inference by using genetic variants as instruments for lifelong differences in metabolic traits, thereby reducing some forms of confounding and reverse causation [14,16,17]. Nevertheless, MR is not equivalent to a randomized nutritional intervention. Instrument strength, horizontal pleiotropy, linkage disequilibrium, ancestry, and the close metabolic correlation of fatty acids within FADS pathways can complicate interpretation. This is particularly relevant when a variant changes several metabolites simultaneously; the estimated effect may represent pathway flux rather than the biological action of a single isolated fatty acid. Larsson et al. reported an association between genetically predicted plasma phospholipid ARA and site-specific cancers, including CRC, supporting a possible role for endogenous n-6 PUFA metabolism [15]. In contrast, broader MR analyses have not consistently supported causal effects for total SFAs or total PUFAs on CRC [18,68]. FADS1/FADS2 are especially important; variants at this locus influence LA, GLA, DGLA, ARA, and several n-3 PUFA intermediates simultaneously [18,19]. Reduced FADS1 activity can increase DGLA while decreasing its conversion to ARA, providing one potential explanation for the inverse association between erythrocyte DGLA and advanced adenomas [28]. Thus, DGLA, ARA, and the ARA/DGLA ratio should be interpreted in relation to desaturase activity rather than as interchangeable n-6 PUFA measures. Host inflammatory phenotype may further modify observed relationships. An MR-based analysis in postmenopausal women suggested interactions between genetically predicted CRP and lifestyle strata including SFA intake [69]. Such analyses are useful for generating effect-modification hypotheses, but they require replication and cannot establish that a particular dietary SFA dose is beneficial or harmful in a defined inflammatory subgroup.

### 3.7. Precursor Lesions, Chemoprevention, and Intervention Evidence

Precursor-lesion and intervention studies provide an essential bridge between observational associations and clinically testable prevention. Conventional adenomas and serrated lesions arise through partly distinct pathways, and an exposure associated with invasive CRC does not necessarily have the same relationship with early lesions. In the prospective erythrocyte analysis by Wang et al., most n-3 and n-6 PUFAs were not strongly associated with conventional adenomas or serrated polyps, although higher DGLA was associated with lower risk of advanced adenomas [26]. That finding is important because it runs counter to a simple interpretation in which all n-6 intermediates are harmful and is compatible with the metabolic distinction between DGLA and ARA. It also illustrates why lesion type, anatomical site, and stage of neoplastic progression should be specified whenever fatty-acid associations are summarized.

Randomized chemoprevention provides a stronger test of whether changing exposure alters a clinically relevant precursor endpoint. The seAFOod Polyp Prevention trial evaluated EPA, aspirin, and their combination in individuals undergoing colorectal adenoma surveillance using a multicenter, randomized, double-blind, placebo-controlled 2 × 2 factorial design [70]. EPA did not significantly reduce the primary endpoint defined by the proportion of participants with one or more adenomas [70]. Secondary analyses, however, suggested heterogeneity in adenoma number, type, and location. These signals should not be elevated above the neutral primary endpoint, but they are useful for hypothesis generation because they suggest that a fatty-acid intervention may not act uniformly across all colorectal lesions. The appropriate interpretation is therefore not simply that EPA “works” or “does not work.” Rather, the seAFOod trial demonstrates that favorable observational n-3 PUFA associations do not automatically translate into a lower primary adenoma-recurrence endpoint when EPA is delivered as a defined intervention [70]. A subsequent secondary analysis examined the *FADS* insertion-deletion variant rs66698963 and suggested a potentially more favorable EPA-associated polyp response in a genetically defined subgroup [71]. This pharmacogenetic or precision-nutrition signal is exploratory and requires external replication, but it creates a coherent link between endogenous fatty-acid metabolism, host genotype, and intervention response.

The same temporal caution applies to molecular subtype analyses. Dietary associations observed among patients with KRAS-mutated tumors characterize heterogeneity among established cancers [54]; they do not show that PUFA exposure generated the *KRAS* mutation or initiated colorectal carcinogenesis. This distinction also corrects the implication that *KRAS* represents an initiating event equivalent to APC, because *KRAS* is classically considered part of adenomatous progression [53,54].

Evidence in patients with established CRC addresses another clinical setting and should be separated from primary prevention and chemoprevention. Meta-analyses of perioperative or adjuvant n-3 PUFA-containing nutritional interventions report improvements in selected immune, inflammatory, nutritional, and recovery-related outcomes [72,73,74,75]. Yue et al. described increases in IgA, IgM, IgG, CD3+, CD4+, and the CD4+/CD8+ ratio, together with improvements in total protein, albumin, and prealbumin [72]. Li L et al. reported fewer postoperative infectious complications in pooled analyses, reductions in TNF-alpha and IL-6, and shorter hospital stay in some preoperative or postoperative comparisons [74]. Liu et al. similarly found reductions in selected inflammatory markers and shorter hospital stays but no consistent effects on CRP, albumin, body weight, overall complications, or CRC mortality across all analyses [75]. The most recent postoperative meta-analysis also reported changes in protein status, lymphocyte subsets, inflammatory markers, and length of hospitalization, while mortality and other oncological outcomes remained non-significant [73]. These results may be clinically useful as supportive-care signals, particularly around surgery, but they do not establish antitumor efficacy, reduced recurrence, or improved survival. Surrogate endpoints should therefore be explicitly separated from hard outcomes.

Some trials use oral EPA and DHA, others use enteral or parenteral lipid emulsions, and some combine n-3 PUFA with arginine, nucleotides, or broader immunonutrition formulations. When an intervention is multicomponent, the observed effect cannot be attributed exclusively to fatty acids. Dose, duration, timing relative to surgery or chemotherapy, baseline nutritional status, comparator, and route of administration also vary substantially [63,72,73,74,75]. Evidence on n-3 PUFA in cancer-associated metabolic support and on non-CRC inflammatory responses should likewise not be interpreted as evidence of CRC-specific antitumor efficacy [76]. Another limitation is that pooled meta-analytic estimates can obscure differences between primary trials in formulation, nutritional indication, operative context, and risk of complications. The randomized crossover comparison of marine n-3 and plant n-6 PUFAs on inflammatory markers provides mechanistic context but is not a CRC-specific efficacy trial [77]. Overall, n-3 PUFA-containing nutritional strategies may improve selected perioperative, inflammatory, immune, or nutritional outcomes in some CRC settings, while evidence remains insufficient for consistent effects on recurrence, progression, mortality, or survival.

### 3.8. Diet-Microbiota-Bile Acid Interactions: Emerging Evidence

Dietary fat can influence the colonic environment through bile-acid metabolism, microbial ecology, epithelial signaling, and interactions with fiber [78,79,80,81,82]. Secondary bile acids, including deoxycholic acid, provide a plausible diet-microbiota link [81,83,84] and MUFA intake has been associated with microbial profiles and selected fecal bile-acid derivatives [85,86]. Prospective evidence has linked a western-style dietary pattern with a higher incidence of CRC containing abundant colibactin-producing pks+ *Escherichia coli*. However, this association was based on an overall dietary pattern characterized by high intake of red and processed meat, sugar, and refined grains, rather than on SFA exposure specifically. Therefore, the available evidence does not establish that SFA independently promotes pks+ *E. coli* colonization or pks+ *E. coli*-associated colorectal carcinogenesis [87]. Current human evidence in this domain is predominantly observational and cannot establish the direction of the diet–microbiota–bile-acid relationship. Associations between fatty-acid intake, microbial composition, and fecal metabolites may also reflect the broader dietary pattern rather than an isolated effect of a particular fatty acid. Consequently, these findings should be considered mechanistically plausible and hypothesis-generating until confirmed in longitudinal and controlled intervention studies.

### 3.9. Integrated Interpretation and Clinical Implications

**Figure 2:**
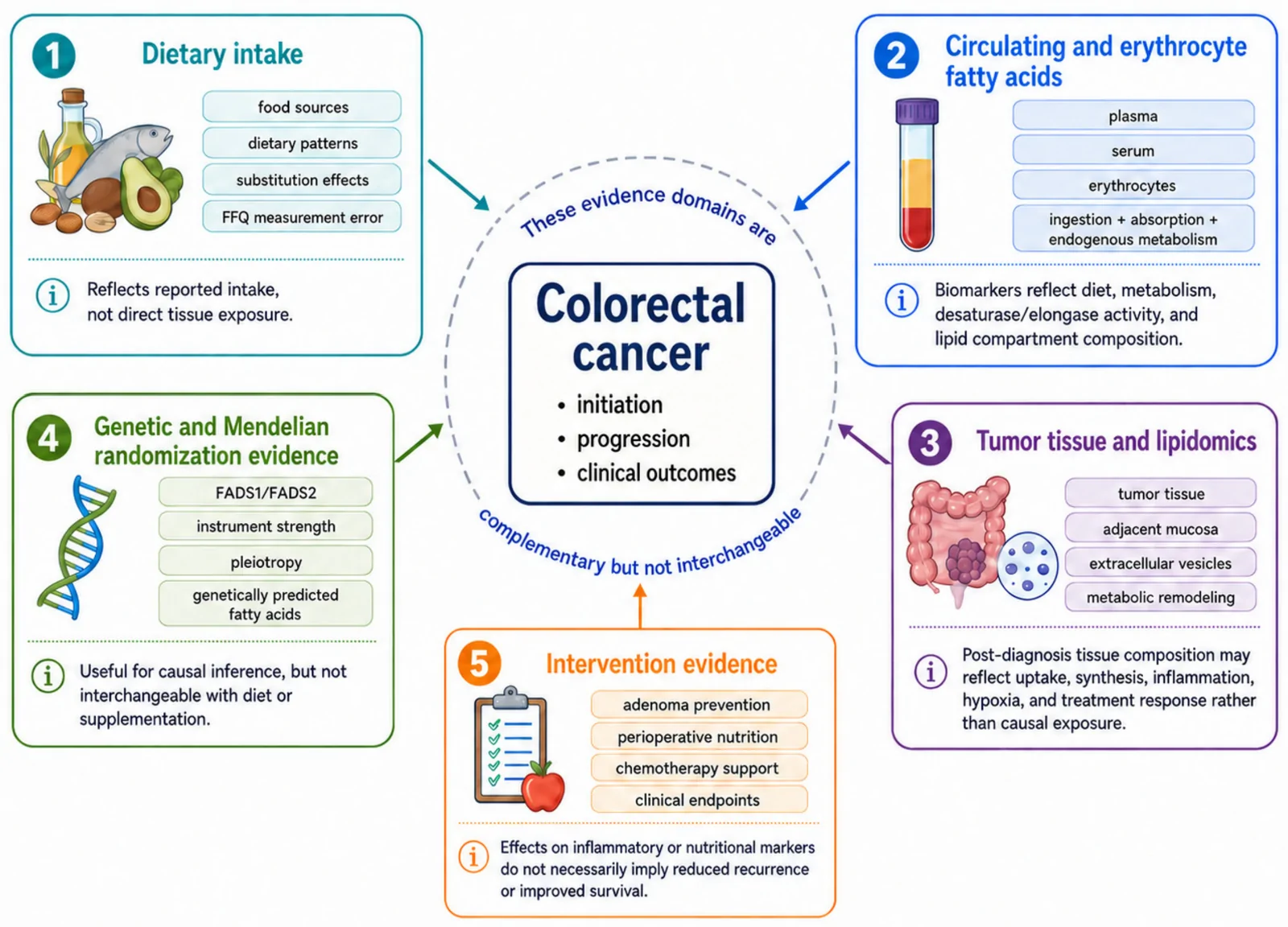
Evidence domains linking fatty acids to colorectal cancer. Overview of the main approaches used to investigate fatty acids in CRC, including dietary intake, circulating and erythrocyte biomarkers, tumor tissue and lipidomics, genetic and Mendelian randomization analyses, and intervention studies. These domains capture distinct aspects of fatty acid exposure and metabolism and should therefore be interpreted as complementary. Abbreviations: CRC, colorectal cancer, FFQ, food frequency questionnaire, FADS1, fatty acid desaturase 1, FADS2, fatty acid desaturase 2.

The integrated framework explains why discordance across studies should not be averaged away. Dietary intake may be null, while plasma is associated with risk because circulating concentrations incorporate absorption and endogenous metabolism; tumor enrichment may reflect selective synthesis or uptake rather than causal exposure, and MR may differ from cohorts because genetic instruments represent lifelong pathway perturbation rather than a dietary dose. Interventions test specified formulations and may alter supportive-care outcomes without changing recurrence or survival (Figure 2). Accordingly, greater interpretive weight should be given to biologically coherent patterns across complementary evidence domains than to the statistical significance of isolated observational findings. Prospective dietary and biomarker studies establish temporality more effectively than retrospective or post-diagnostic analyses, but they remain susceptible to confounding, measurement error, and underlying metabolic determinants of exposure. Observational findings should therefore be interpreted as evidence of association and biological coherence. Causal interpretation can be strengthened through triangulation with genetic and other causal-inference approaches, whereas clinical recommendations ultimately require adequately designed randomized interventions with clinically relevant endpoints. The evidence therefore changes how fatty acids classes should be summarized. Dietary SFA associations are inconsistent and strongly influenced by food source, substitution, and dietary pattern [35,36,39,40,41,42,43,45]. Total MUFA intake has no consistent independent association with CRC, but endogenous MUFA metabolism and tissue distribution can be altered in tumors [35,36,45,52]. n-6 PUFAs cannot be classified uniformly as harmful because LA, DGLA, and ARA occupy different metabolic positions and circulating n-6 may show neutral or inverse associations [18,19,25,26,51,58]. n-3 PUFA evidence is comparatively favorable for selected dietary and circulating exposures [29,48,49,50,52,57,58] (Table 1), yet randomized prevention and clinical-intervention evidence is more limited and outcome-specific [70,71,72,73,74,75]. Clinically, current evidence does not justify prescribing one fatty acid, a universal supplement dose, or a target n-6/n-3 PUFA ratio for CRC prevention or treatment. Counseling should prioritize dietary quality, plant foods and fiber, appropriate fish sources, and limitation of red and processed meat within established prevention guidance [3,4,5,42]. Future studies should connect repeated dietary assessment with objective biomarkers, metabolic genotype, tumor subsite and molecular phenotype, and randomized oncological endpoints. In this context, precision nutrition should be considered a research framework rather than a currently established clinical strategy for CRC. Future fatty-acid interventions could potentially be stratified according to genetic variation in fatty-acid metabolism (e.g., *FADS1/FADS2* and *ELOVL*-related variants), circulating or erythrocyte fatty-acid profiles, desaturase-related metabolic phenotypes, tumor molecular characteristics, metabolic status, and potentially gut-microbiome features. However, these characteristics have not yet been prospectively validated as treatment-selection biomarkers, and therefore they cannot currently be used to recommend individualized EPA/DHA doses, specific fatty-acid targets, or personalized n-6/n-3 ratios. The main findings and their current translational interpretation are summarized in Table 3.

**Table 1:** Selected observational, biomarker, tissue, and genetic human evidence on fatty acids and colorectal cancer.

Author, Year	Objective, Exposure/Intervention, Study Design	Duration	n	Results
Larsson S et al. (2020) [15]	Evaluate whether genetically predicted plasma phospholipid arachidonic acid (ARA) concentrations are associated with the risk of 10 site specific cancers, including CRC, using genetic variants associated with ARA levels. (Mendelian randomization)	NA	367,643	Higher genetically predicted plasma phospholipid ARA concentrations were associated with increased CRC risk (OR per SD = 1.08; 95% CI: 1.05–1.11; *p* = 6.3 × 10^−8^). The association was consistent across genetic instruments and data sources, supporting a possible role of endogenous ARA metabolism in CRC.
Kim E et al. (2021) [56]	Assess the association between red or processed meat consumption and CRC risk in a Korean population. (Case-control study)	NA	30 (Cases = 15)	Red or processed meat consumption was not higher in cases than in controls. However, most of the 31 measured fatty acids, including several SFA, were higher in cases than in controls. Cases also showed upregulation of PTGS2, which is involved in pro-inflammatory lipid mediator pathways.
Jung SY et al. (2021) [69]	Investigate the genetically predicted CRP phenotype as a potential causal factor in primary CRC risk among postmenopausal women. (MR with lifestyle interaction)	NA	10,142 (Cases = 737)	The analysis suggested a potential causal association between lifelong exposure to CRP and CRC risk, particularly among women with low SFA intake. In lifestyle-stratified analyses, genetically predicted CRP was associated with higher CRC risk in the low-SFA subgroup and lower risk in the high-SFA subgroup.
Nguyen S et al. (2021) [35]	Evaluate the association between dietary fatty-acid intake and CRC risk among Chinese men and additionally perform a meta-analysis of recent evidence. (Prospective cohort + meta-analysis)	9.8 years	59,986 (Cases = 876)	Dietary SFA and MUFA intakes were not significantly associated with CRC risk (HR = 0.92; 95% CI: 0.74–1.14; *p* = 0.47). Consistently, the me-ta-analysis confirmed an overall null association between total dietary fatty-acid intake and CRC risk in men.
Bestard-Escalas J, et al. (2021) [66]	Investigate the unsaturation of plasma extracellular vesicles as a potential source of non-invasive biomarkers for CRC. (Observational lipidomic study)	NA	62	The relative proportion of 34:1 species was significantly lower in diseased groups, suggesting a shift in the exosomal lipid profile from monounsaturated species toward more polyunsaturated species, such as C38:4. This pattern represents an exploratory discriminative lipidomic signature requiring external validation.
T Juloski J et al. (2021) [61]	Investigate differences in fatty-acid content between CRC tissue and adjacent healthy intestinal tissue in adult and older patients of both sexes. (Paired tumor-adjacent tissue study)	NA	52	Stearic acid (C18:0) and total SFAs were higher in tumor tissue than in adjacent mucosa. The saturation index was considered an exploratory tissue signature requiring external validation. Oleic acid (C18:1n-9) and total MUFAs were significantly higher in adjacent non-tumor tissue than in tumor samples. Overall PUFA differences were not interpreted as evidence of CRC risk. DHA, total n-3 PUFA, and the n-6/n-3 ratio differed between tumor and adjacent mucosa; these post-diagnostic differences do not establish causality.
Wang L et al. (2021) [26]	Examine the associations between erythrocyte PUFA levels, including both n-3 and n-6 fatty acids, and the risk of colorectal serrated polyps and conventional adenomas. (Prospective cohort)	20 years	4517	No n-3 or n-6 PUFA showed statistically significant associations with the overall risk of conventional adenomas or serrated polyps. Higher DGLA (20:3n-6) was associated with a lower risk of advanced adenomas in selected analyses.
Tu K et al. (2022) [43]	Examine whether dietary-derived fatty-acid patterns were associated with CRC risk in a Chinese population. (Case-control study)	July 2010 to May 2021	5612 (Cases 2806)	A significant inverse association was observed between the medium- and long-chain SFA pattern and CRC risk (aOR = 0.34; 95% CI: 0.27–0.42).
El Asri A et al. (2022) [54]	Explore the associations between fat intake and *KRAS* mutations in codons 12 and 13 in cases of CRC in the Moroccan population. (Case-only molecular epidemiology)	September 2009 to February 2017	151	No statistically significant associations were observed between total fat or SFA intake and *KRAS* mutations. Higher MUFA intake (>34.9 g/day) was initially associated with *KRAS* mutation status (OR = 1.56), but the association was no longer statistically significant after adjustment for con-founding factors. High PUFA intake (>16.9 g/day) was associated with a greater likelihood of *KRAS* mutations compared with low PUFA intake (≤16.9 g/day) (aOR = 2.15; *p* < 0.05).
Hoang T et al. (2022) [85]	Investigate associations between diet and gut-microbiota diversity and taxonomic composition in patients with CRC. (Cross-sectional observational analysis)	NA	115	Higher MUFA intake was associated with distinct gut microbiota profiles (*p* < 0.01) in colorectal cancer patients, with specific bacterial taxa differentially enriched according to consumption levels. These findings suggest that habitual MUFA intake may modulate microbial composition in CRC.
Yiannakou I et al. (2022) [36]	Investigate prospectively assessed intakes of processed and unprocessed red meat and fatty acids in relation to CRC risk using data from the Black Women’s Health Study. (Prospective cohort)	(BWHS 1995–2018)	52,695	Unprocessed red meat intake was associated with a 33% higher CRC risk per 100 g/day (HR = 1.33; 95% CI: 1.03–1.71) and with a significantly higher risk of late-onset CRC (≥50 years; HR = 1.41; 95% CI: 1.05–1.88). Processed red meat and total SFA and MUFA intakes were not associated with CRC risk.
Shekari S, et al. (2022) [51]	Evaluate associations between habitual dietary intake of different fatty acids, assessed using dietary questionnaires, and CRC risk, including PUFAs, LA, and ALA. (Case-control study)	2020–2021	480 (Cases = 160)	CRC cases had a higher mean intake of total PUFA than controls (16.54 ± 4.20 vs. 15.41 ± 4.44 g/day; *p* = 0.012). Higher dietary intakes of LA (OR = 0.85; *p* = 0.001) and ALA (OR = 0.75; *p* = 0.04) were inversely associated with CRC risk after multivariable adjustment.
Lu Y et al. (2023) [52]	Synthesize evidence on associations of dietary intake (28 FA) and blood levels (18 FA) with CRC risk through meta-analysis of observational and Mendelian-randomization studies. (Meta-analysis)	NA	2495,256 (Cases = 52,980) 7570 (Cases = 2086)	Dietary SFAs showed a modest inverse pooled association (OR = 0.91), while blood SFAs showed no significant association with CRC risk; this does not establish class-wide protection. Total dietary MUFA intake was not significantly associated with colorectal cancer risk. The pooled analysis showed no statistically significant effect. Higher dietary EPA (RR = 0.83), DHA (RR = 0.80), and n-3 DPA (RR = 0.75) were associated with lower CRC risk; the n-6/n-3 ratio should not be treated as a universal causal indicator.
Wu Q et al. (2023) [55]	Investigate the association of serum SFA and very-long-chain SFA (VLCSFA) with CRC risk in a Chinese population. (Case-control study)	July 2010 to May 2021	1360 (Cases = 680)	Total SFAs were positively associated with CRC risk (adjusted OR, quartile 4 vs. quartile 1 = 2.64), whereas VLCSFA were inversely associated with CRC risk (adjusted OR, quartile 4 vs. quartile 1 = 0.51). These findings indicate that higher total serum SFAs and lower serum VLCSFA were associated with increased CRC risk in this population.
Wang J, et al. (2023) [67]	Evaluate whether genetic variants in lipid-metabolism-related genes, including *FASN*, were associated with progression-free survival and treatment response in patients with metastatic CRC receiving bevacizumab-based chemotherapy. (Retrospective cohort)	2007–2015	968	Genetic variants in lipid-metabolism pathways may influence treatment response in metastatic CRC. Polymorphisms in *FASN*, a key enzyme involved in fatty-acid synthesis, were associated with shorter progression-free survival in patients treated with bevacizumab (multivariable HRs ≈ 2.87 and ≈2.07).
Haycock PC et al. (2023) [18]	Investigate associations between circulating fatty acid profiles, including PUFA (n-3 and n-6), and CRC risk in European populations using prospective data and Mendelian randomization. (Mendelian randomization)	Follow up > 10 years	150,000	No consistent, robust links between total PUFA levels and CRC risk after multivariable adjustment. MR analyses did not support a causal relationship between genetically instrumented PUFA levels (n-3 or n-6) and CRC risk.
Kato A, et al. (2023) [48]	Evaluate associations of total n-3 PUFAs, marine-derived n-3 PUFAs (EPA, n-3 DPA, DHA) and ALA as plant-derived n-3 PUFA with the risk of CRC by subsite in the Japan Collaborative Cohort Study. (Prospective cohort)	13.8 years	42,536 (Cases = 699)	A significant inverse association was observed between dietary ALA intake and distal colon cancer risk. Participants in the highest intake quartile had a 59% lower risk (HR = 0.41; 95% CI: 0.21–0.81; *p* for trend = 0.01). Marine-derived n-3 PUFA intake was not significantly associated with overall CRC risk or with risk by anatomical subsite.
Bhatt K, et al. (2023) [65]	Investigate differences in serum metabolite and lipid profiles, among individuals with colorectal adenocarcinoma, adenoma, and healthy controls, and assess their potential utility as biomarkers for early CRC detection using advanced lipidomics and metabolomics analysis. (Observational cross-sectional)	NA	64	Lipidomic profiling identified eight fatty acids that significantly distinguished CRC from adenoma and control groups, including four n-3 PUFAs (EPA, C20:4n-3, n-3 DPA, DHA) and three n-6 PUFAs (C18:3n-6, C20:3n-6, C22:5n-6). n-3 PUFAs were generally lower in the CRC group, whereas n-6 PUFAs tended to be higher in CRC cases than in adenoma and control groups. These findings are exploratory and require external validation.
Skulsky SL et al. (2024) [41]	Investigate whether dietary patterns previously associated with type 2 diabetes, cardiovascular disease, and all-cause mortality are also associated with CRC. (Prospective cohort)	8 years	114,443 (Cases = 1089)	DP1, characterized by high consumption of chocolate, confectionery, butter, low-fiber bread, red and processed meats, and alcohol, and low intake of fruits, vegetables, and whole grains, was positively associated with colorectal cancer risk. After adjustment for confounders, individuals in the highest quintile had increased risk of overall CRC (HR = 1.34, Ptrend = 0.005) and rectal cancer (HR = 1.58; Ptrend = 0.009).
Poorolajal J et al. (2024) [37]	Investigate associations of red meat, processed meat, and fish consumption with the risk of gastrointestinal cancers. (Systematic review and meta-analysis)	NA	5,749,219 (Studies = 95)	The consumption of high levels of red meat and processed meat, as compared to low levels, was found to significantly increase the risk of developing several gastrointestinal cancers including colon, rectal and colorectal. In contrast, the consumption of high levels of fish, compared to low levels, significantly reduced the risk of colon, rectal, and colorectal cancers.
Jiang D et al. (2025) [68]	Investigate the potential causal relationships between fatty acids and CRC by leveraging data from the UK Biobank. Eighteen fatty acid–related phenotypes were evaluated, and single-nucleotide polymorphisms were employed as instrumental variables within a Mendelian randomization framework. (Mendelian randomization)	NA	19,948 (Cases = 7824)	No significant associations were found between CRC and SFAs.
Sassano M et al. (2025) [40]	Evaluate the association between dietary patterns (DP) and colorectal cancer (CRC) in an Iranian population. (Multicenter case-control study)	2017–2022	3204 (Cases = 865)	A Western-type dietary pattern was strongly associated with increased CRC risk (OR__Q4vsQ1_ = 4.15; 95% CI: 2.49–6.90; Ptrend < 0.001). Likewise, a dietary pattern high in unhealthy fats, characterized by elevated intake of animal products, was positively associated with CRC (OR__Q4vsQ1_ = 2.14; 95% CI: 1.40–3.26; Ptrend < 0.001).
Mombert P et al. (2025) [44]	Analyze the nutritional risks and modeled health benefits associated with greater adherence to plant-based dietary patterns in a subsample of the French population. (Dietary modeling study)	NA	1365	Fewer participants following plant-based dietary patterns exceeded the upper intake limit for SFAs. Modeling suggested approximately 132,700 DALYs averted (95% uncertainty interval: approximately 125,400–140,000), with a larger modeled benefit in males than in females; CRC contributed to the estimated health benefit.
Rothbard SM, et al. (2025) [50]	Evaluate whether fish intake and PUFA consumption, particularly marine n-3 PUFA, are associated with CRC risk in the Black Women’s Health Study. (Prospective cohort)	24 years (1995–2021)	52,690 (Cases = 687)	High baked-fish intake (>8.09 g/1000 kcal vs. <0.38 g/1000 kcal) was associated with reduced overall CRC risk (HR: 0.74). High n-3 PUFA intake was associated with lower proximal colon cancer risk (HR: 0.61). Total PUFA intake was not significantly associated with overall CRC risk.
Wolf PG, et al. (2025) [86]	Examine associations between dietary nutrients known to modulate bile acids, including MUFAs, and fecal concentrations of bile-acid derivatives that may influence CRC risk through bacterial metabolism in the colon. (Cross-sectional observational analysis)	NA	138	MUFA intake was inversely associated with several abundant DCA derivatives in regression models, indicating that higher MUFA consumption was linked to lower levels of specific secondary bile acid metabolites with potential carcinogenic properties.
Zhang Y et al. (2025) [62]	Investigate associations of plasma n-6 and n-3 PUFA percentages and the n-6/n-3 ratio with the incidence of overall and 19 site-specific cancers in UK Biobank. (Prospective population-based cohort)	12.9 years	253,138 (29,838 incident cancers)	Higher plasma n-6 PUFA percentages were inversely associated with colon cancer (HR per SD = 0.94; 95% CI: 0.91–0.97) and rectal cancer (HR = 0.95; 95% CI: 0.91–0.99) in the main models. Plasma n-3 PUFA percentages were also inversely associated with colon cancer (HR = 0.96; 95% CI: 0.92–0.99) but not clearly with rectal cancer. After additional site-specific adjustment, the inverse association with colon cancer remained for both n-6 and n-3 PUFAs.
Aldoori J et al. (2025) [57]	Examine associations between baseline plasma total n-3 PUFA and DHA concentrations and incident CRC risk, including analyses by tumor location, sex, and nonlinear dose-response relationships in UK Biobank. (Prospective cohort)	13.4 years	234,598 (Cases = 2602)	Higher plasma total n-3 PUFA and DHA concentrations were modestly inversely associated with CRC risk. For total n-3 PUFAs, T2 HR = 0.88 (95% CI: 0.80–0.97) and T3 HR = 0.91 (95% CI: 0.83–1.00) versus T1. For DHA, T2 HR = 0.89 (95% CI: 0.80–0.98) and T3 HR = 0.91 (95% CI: 0.82–1.00). The DHA association was nonlinear, with evidence of a plateau at higher plasma concentrations, and was more pronounced for proximal colon cancer and among men.

Studies were selected for inclusion in the tables when they represented key human evidence discussed in the narrative and contributed directly to the principal conclusions of the review; the tables are not intended to be exhaustive. Abbreviations: CRC, colorectal cancer, FA, fatty acid, SFA, saturated fatty acid, MUFA, monounsaturated fatty acid, PUFA, polyunsaturated fatty acid, EPA, eicosapentaenoic acid, DHA, docosahexaenoic acid, DPA, docosapentaenoic acid, KRAS, Kristen rat sarcoma virus gene, CRP, C-reactive protein, PTGS2, prostaglandin-endoperoxide synthase 2, DP, dietary pattern, VLCSFA, very long-chain saturated fatty acids, DALYs, disability-adjusted life years, FASN, fatty acid synthase, DCA, deoxycholic acid, DGLA, dihomo-gamma-linolenic acid, LA, linoleic acid, ALA, alpha-linolenic acid.

**Table 2:** Selected Clinical and Intervention Evidence in Colorectal Cancer.

Author, Year	Objective-Exposure/Intervention –Study Desing	Duration	n	Results
Hull MA et al. (2018) [70]	Evaluate the chemopreventive efficacy of EPA 2 g/day, aspirin 300 mg/day, alone or in combination, compared with placebo in individuals at high risk of colorectal adenoma recurrence undergoing surveillance colonoscopy. (Multicenter, randomized, double-blind, placebo-controlled, 2 × 2 factorial trial)	12 months	709	EPA did not significantly reduce the primary endpoint, defined as the proportion of participants with ≥1 adenoma at surveillance colonoscopy (RR = 0.98; 95% CI: 0.87–1.12; *p* = 0.81). However, secondary analyses showed a modest reduction in conventional adenoma number (IRR = 0.86; 95% CI: 0.74–0.99) and left-sided adenoma number (IRR = 0.75; 95% CI: 0.60–0.94) with EPA.
Yue T et al. (2022) [72]	Evaluate n-3 PUFA supplementation compared with conventional nutrition or blank control in perioperative or postoperative patients with CRC. (Meta-analysis)	NA	1613	n-3 PUFA supplementation significantly increased IgA (SMD = 0.54), IgM (SMD = 0.52), IgG (SMD = 0.65), CD3+ (SMD = 0.73), CD4+ (SMD = 0.76), and the CD4+/CD8+ ratio (SMD = 0.66). CD8+ cells decreased (SMD = −0.28). Nutritional status also improved, as reflected by total protein (SMD = 0.53), albumin (SMD = 0.43), and prealbumin (SMD = 0.46).
Li L et al. (2023) [74]	Investigate the effects of PUFA supplementation on postoperative complications, immune regulation, inflammatory markers, body weight, and BMI in patients with CRC. Assessed outcomes included postoperative complications, the CD4/CD8 ratio, albumin, and CRP levels. (Meta-analysis)	NA	702 (12 RCT)	PUFA supplementation significantly reduced total postoperative infectious complications (RR = 0.37; *p* = 0.02) and shortened hospital stay in both preoperative (WMD = −2.27; *p* < 0.001) and postoperative settings (WMD = −2.66; *p* = 0.01). It also decreased TNF-α (SMD = −0.56; *p* = 0.007) and IL-6 (SMD = −0.54; *p* = 0.004). Among patients receiving chemotherapy, PUFA supplementation increased albumin (WMD = 0.48; *p* = 0.03) and reduced CRP (WMD = −6.12; *p* = 0.02).
Liu H, et al. (2023) [75]	Evaluate the efficacy of n-3 PUFA as adjuvant treatment in patients with CRC undergoing surgery with chemotherapy or surgery alone. (Meta-analysis)	NA	1556 (19 RCT)	No significant effect was observed on overall infectious and noninfectious complications (RR = 0.96; 95% CI: 0.65–1.42), CRC mortality, body weight, or albumin. Selected inflammatory markers and hospital stay improved in some analyses.
Sun G et al. (2024) [71]	Determine whether the *FADS* insertion-deletion polymorphism rs66698963 modifies the response to EPA supplementation for colorectal polyp prevention in a secondary analysis of the seAFOod trial. Participants received EPA 2 g/day or placebo within the original factorial trial. (Secondary pharmacogenetic analysis of a randomized con-trolled trial)	12 months	528	EPA use irrespective of genotype was not associated with a significant reduction in total colorectal polyp number (IRR = 0.92; 95% CI: 0.74–1.16). However, among participants carrying ≥ 1 insertion (I) allele, EPA was associated with a significant reduction in colorectal polyp number (IRR = 0.50; 95% CI: 0.28–0.90), with similar findings for polyp detection rate.
Li H, et al. (2026) [73]	Evaluate the effects of n-3 PUFAs (EPA, DHA, and/or LA) on postoperative recovery, immune function, inflammation, and clinical outcomes in patients with CRC undergoing surgery. (Systematic review and meta-analysis)	NA	2889 (34 RCT)	Supplementation significantly improved total protein levels (*p* < 0.00001), CD3+, CD4+, and CD8+ T-cell levels, and the CD4+/CD8+ ratio (all *p* < 0.0001), as well as KPS (*p* = 0.04). CRP, IL-6, TNF-α, and procalcitonin were significantly reduced (*p* = 0.004 to <0.00001), and hospital stay was significantly shorter (*p* < 0.00001). No significant effects were observed on white blood cell count, transcription factor activity, CRC mortality, or crypt proliferation indices.

Studies were selected for inclusion in the tables when they represented key human evidence discussed in the narrative and contributed directly to the principal conclusions of the review; the tables are not intended to be exhaustive. Abbreviations: CRC, colorectal cancer, PUFA, polyunsaturated fatty acid, IgA, immunoglobulin A, IgM, immunoglobulin M, IgG, immunoglobulin G, CD3+, cluster of differentiation 3, CD8+, cluster of differentiation 8, BMI, body mass index, CD4+, cluster of differentiation 4, CRP, C-reactive protein, TNF-alpha, tumor necrosis factor alpha, IL-6, interleukin 6, EPA, eicosapentaenoic acid, DHA, docosahexaenoic acid, LA, linoleic acid.

**Table 3:** Narrative summary of the current translational interpretation of fatty-acid evidence in colorectal cancer.

Primary Finding	Evidence Basis	Current Evidence Status	Clinical Interpretation
Selected n-3 biomarkers show inverse CRC associations	Prospective cohorts and meta-analyses	Suggestive, not validated	Does not define a supplementation dose or target concentration
Tumor fatty-acid remodeling	Tumor-tissue and lipidomic Studies	Exploratory	Reflects metabolic remodeling; does not establish etiologic exposure
FADS-related modification of EPA response	Secondary pharmacogenetic analysis	Exploratory	Requires prospective replication before genotype-guided intervention
Perioperative n-3 effects	Randomized trials and meta-analyses	Supportive care evidence	May improve selected inflammatory, immune, nutritional, or recovery outcomes, does not establish antitumor efficacy
EPA for adenoma prevention	Randomized chemoprevention trial	Neutral primary endpoint, secondary signals	Does not support routine EPA chemoprevention
Specific EPA/DHA dose or n-6/n-3 target	Current human evidence	Not established	No specific dose or ratio can currently be recommended

## 4. Limitations and Challenges

This review has several limitations. As a structured narrative review, it was designed to integrate heterogeneous domains of human evidence rather than to provide a systematic quantitative synthesis or pooled effect estimate. The primary search focused on recent literature, while selected earlier studies were included when they were considered pivotal or necessary to contextualize the evidence; therefore, some relevant studies may not have been captured. In addition, the marked heterogeneity in study design, fatty-acid assessment, biological matrices, populations, and clinical endpoints limits direct comparison across studies. No formal risk-of-bias or certainty-of-evidence assessment was carried out. Likewise, a formal classification of findings according to predefined levels of evidence or translational relevance was not conducted, consistent with the structured narrative design of this review, and may introduce some subjectivity into the interpretation of results. Evidence was instead interpreted according to study design, temporality, susceptibility to confounding or reverse causation, and the clinical relevance of the endpoint.

The evidence included in this review also has domain-specific limitations, summarized in Table 4. Dietary studies remain susceptible to measurement error and residual confounding; biomarker studies integrate dietary and endogenous metabolic influences; tumor-tissue studies are mainly post-diagnostic; and Mendelian-randomization analyses are constrained by pleiotropy and instrument validity. Intervention studies are further limited by heterogeneity in formulation, dose, route, timing, and the frequent use of surrogate rather than oncological endpoints.

Future research should prioritize longitudinal and integrative designs that combine repeated dietary assessment with circulating or erythrocyte biomarkers, genetic and metabolic phenotyping, and tumor characteristics. Adequately powered randomized trials using clinically relevant endpoints, such as adenoma recurrence, cancer recurrence, progression, and survival, are particularly needed before specific fatty-acid interventions, doses, or target n-6/n-3 ratios can be recommended.

## 5. Conclusions

The relationship between fatty acids and CRC cannot be reduced to a model in which SFAs and n-6 PUFAs are harmful, MUFAs are neutral, and n-3 PUFAs are protective. Associations depend on the individual fatty acid, food source, dietary substitution, biological matrix, endogenous synthesis and desaturation, anatomical site, sex, disease stage, and study design. Dietary studies primarily inform food-related exposure; circulating and erythrocyte biomarkers integrate diet with metabolism; tumor tissue describes post-diagnostic metabolic remodeling; MR interrogates lifelong genetically influenced pathways; and interventions test specific formulations in defined clinical settings. These domains are complementary but not interchangeable. Current evidence is compatible with potentially favorable associations for selected n-3 PUFA exposures and with supportive perioperative effects of some n-3-PUFA-containing formulations, but it does not support universal supplementation for CRC prevention, recurrence reduction, or survival improvement. Similarly, available human evidence does not justify class-wide causal statements for SFAs, MUFAs, or n-6 PUFAs, nor use of the n-6/n-3 PUFA ratio as a universal risk indicator. Future research should combine repeated dietary assessment with objective biomarkers, FADS/ELOVL-related metabolic phenotyping, tumor molecular characteristics, and adequately powered randomized trials with oncological endpoints. Until such evidence is available, nutritional recommendations should emphasize healthy dietary patterns and high-quality food sources.

## Figures and Tables

**Table 4:** Strengths, limitations, and interpretative boundaries of complementary fatty-acid evidence domain in colorectal cancer.

Evidence Domain	Primary Construct Assessed	Main Strengths	Main Limitations	Appropriate Interpretation
Dietary intake	Food-related FA exposure	Prospective evaluation, directly relevant to dietary recommendations	FFQ measurement error, residual confounding, food-matrix effects, cooking methods, and substitution effects	Association with dietary exposure, not tissue FA availability
Plasma/serum FA	Integrated dietary exposure, absorption, and metabolism	Objective measurement	Shorter exposure window, lipid-fraction dependence, endogenous synthesis, and reverse causation	Metabolic biomarker rather than direct dietary measure
Erythrocyte FA	Longer-term integrated FA status	More stable than plasma	Desaturation/elongation; membrane turnover; compositional data	Longer-term metabolic phenotype
Tumor tissue/lipidomics	Tumor-associated lipid remodeling	Direct assessment of tumor biology	Post-diagnostic sampling, reverse causation, tumor heterogeneity, and small sample sizes	Metabolic adaptation, not etiologic exposure
Genetic/MR	Lifelong genetically influenced FA metabolism	Reduced susceptibility to conventional confounding and reverse causation	Pleiotropy, weak instruments, correlated FADS metabolites, and ancestry-related limitations	Causal-pathway inference, not equivalent to supplementation
Adenoma/chemoprevention trials	Modification of precursor lesions	Randomization; defined intervention	Endpoint heterogeneity, subgroup findings, and limited generalizability	Stronger evidence for specific precursor outcomes
Perioperative/CRC interventions	Response to defined nutritional intervention	Experimental manipulation in patients	Heterogeneity in dose, formulation, route, multicomponent interventions, and surrogate endpoints	Supportive-care efficacy, not necessarily antitumor efficacy
Microbiome/bile acids	Host–diet–microbial interactions	Mechanistic integration	Predominantly observational/cross-sectional; interindividual variability	Emerging hypothesis, generating evidence

Abbreviations: CRC, colorectal cancer, FA, fatty acid, FFQ, food frequency questionnaire, MR, Mendelian randomization, FADS, fatty acid desaturase.

## Data Availability

No new data were created or analyzed in this study. Data sharing is not applicable to this article.

## Abbreviations

LA	Linoleic acid (C18:2n-6)
ARA	Arachidonic acid (C20:4n-6)
ALA	Alpha-linolenic acid (C18:3n-3)
GLA	gamma-linolenic acid (18:3n-6)
DGLA	dihomo-gamma-linolenic acid (20:3n-6)
n-3 DPA	n-3 docosapentaenoic acid (22:5n-3)
CRC	Colorectal cancer
DHA	Docosahexaenoic acid (C22:6n-3)
EPA	Eicosapentaenoic acid (C20:5n-3)
FA	Fatty acid
MR	Mendelian randomization
MUFA	Monounsaturated fatty acid
PUFA	Polyunsaturated fatty acid
SFA	Saturated fatty acid
